# Crystal structure of 1,5-diethyl-1*H*-1,5-benzodiazepine-2,4(3*H*,5*H*)-di­thione

**DOI:** 10.1107/S205698901402790X

**Published:** 2015-01-03

**Authors:** Abderrahman Lamkaddem, Mohamed Harcharras, Abdelillah Shaim, Hafid Zouihri, Bousselham Echchahed, Wenhua Bi

**Affiliations:** a Laboratoire de Synthèse Organique et des Procédés Éxtraction, Université Ibn Tofail, Faculté des Sciences, 14000 Kénitra, Morocco; b Laboratoire de Synthèse Organique et Organométallique et Théorique, Université Ibn Tofail, Faculté des Sciences, 14000 Kénitra, Morocco; c Laboratoire de Physico-chimie du Solide, Université Ibn Tofail, Faculté des Sciences, 14000 Kénitra, Morocco; d Centre Universtaire d’Analyse, d’Expertise, de Transfert de Technologie et d’Incubateur, Université Ibn Tofail, BP 242, Kénitra, Morocco; e Laboratoire d’Electrochimie, Corrosion et Environnement, Université Ibn Tofail, Faculté des Sciences, 14000 Kénitra, Morocco; f Département de Chimie, Université Laval, Québec, QC, G1V 0A6, Canada

**Keywords:** crystal structure, benzodiazepine, boat conformation.

## Abstract

In the title compound, C_13_H_16_N_2_S_2_, the seven-membered ring adopts a boat conformation, with the two phenyl­ene C atoms representing the stern and the methyl­ene C atom as the prow. The thione S atoms and N-bound ethyl groups lie on the opposite side of the mol­ecule to the phenyl­ene ring so that the mol­ecule approximates mirror symmetry. In the crystal, supra­molecular layers in the *bc* plane are sustained by a pair of C—H⋯S inter­actions to the same S atom acceptor.

## Related literature   

For the biological activity of benzodiazepine derivatives, see: Kumar *et al.* (2006 ▸); Swamy *et al.* (2008 ▸). For a related structure, see: Ourahou *et al.* (2010 ▸).
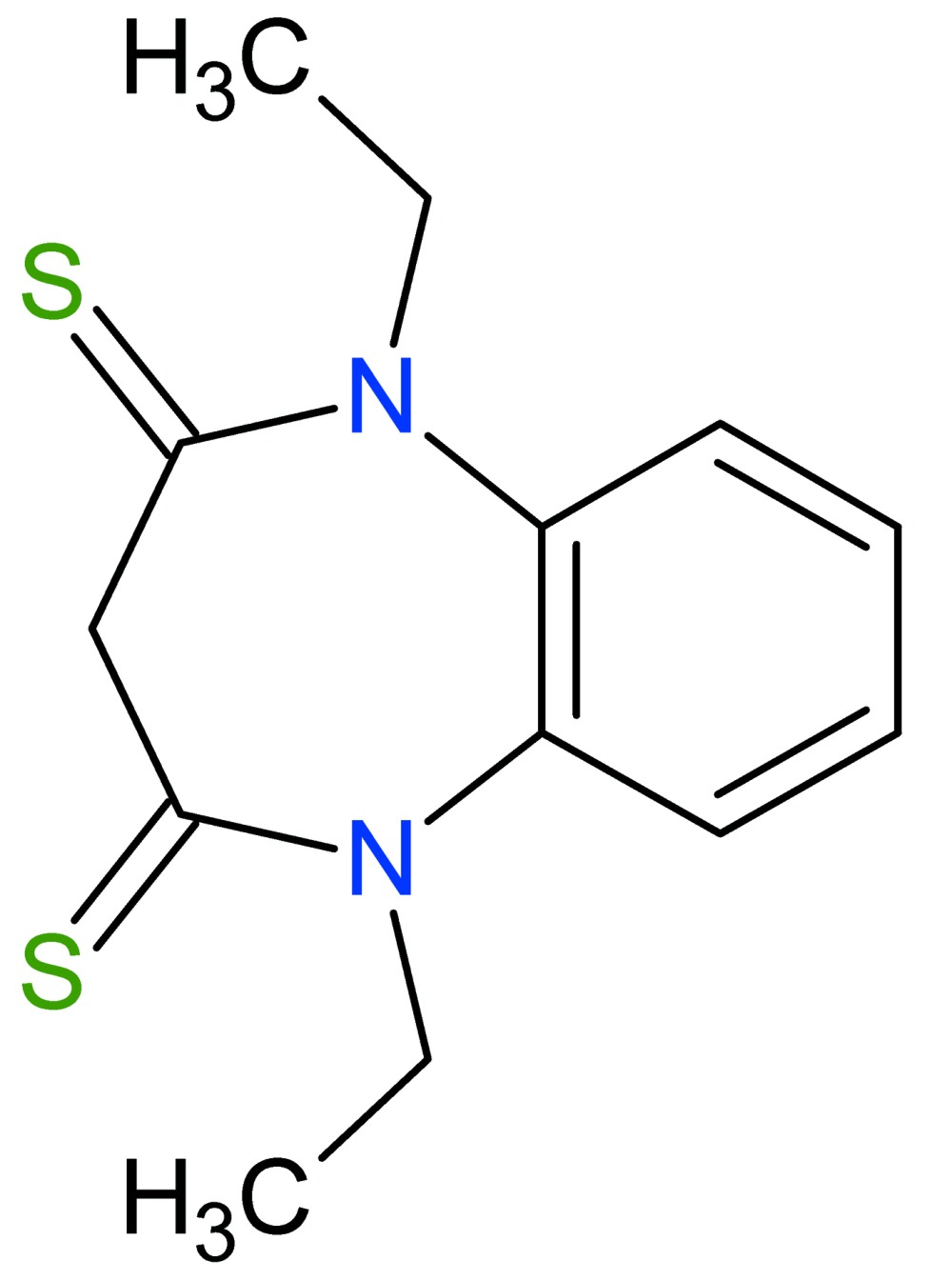


## Experimental   

### Crystal data  

C_13_H_16_N_2_S_2_
*M* *_r_* = 264.40
Monoclinic, 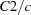

*a* = 19.8896 (2) Å
*b* = 8.8743 (1) Å
*c* = 15.5361 (2) Å
β = 104.087 (1)°
*V* = 2659.75 (5) Å^3^
*Z* = 8
Mo *K*α radiation
μ = 0.38 mm^−1^
*T* = 150 K
0.44 × 0.28 × 0.26 mm

### Data collection  

Bruker APEXII CCD area-detector diffractometer
Absorption correction: multi-scan (*SADABS*; Bruker, 2009 ▸) *T*_min_ = 0.880, *T*_max_ = 0.906
14634 measured reflections
3312 independent reflections
2963 reflections with *I* > 2σ(*I*)
*R*_int_ = 0.022

### Refinement  

*R*[*F* ^2^ > 2σ(*F* ^2^)] = 0.029
*wR*(*F* ^2^) = 0.083
*S* = 1.05
3312 reflections
154 parameters
H-atom parameters constrained
Δρ_max_ = 0.39 e Å^−3^
Δρ_min_ = −0.21 e Å^−3^

### 

Data collection: *APEX2* (Bruker, 2009 ▸); cell refinement: *SAINT-Plus* (Bruker, 2009 ▸); data reduction: *SAINT-Plus*; program(s) used to solve structure: *SHELXS97* (Sheldrick, 2008 ▸); program(s) used to refine structure: *SHELXL97* (Sheldrick, 2008 ▸); molecular graphics: *PLATON* (Spek, 2009 ▸); software used to prepare material for publication: *publCIF* (Westrip, 2010 ▸).

## Supplementary Material

Crystal structure: contains datablock(s) I. DOI: 10.1107/S205698901402790X/tk5353sup1.cif

Structure factors: contains datablock(s) I. DOI: 10.1107/S205698901402790X/tk5353Isup2.hkl

Supporting information file. DOI: 10.1107/S205698901402790X/tk5353Isup3.cml

**Fig. 1.**
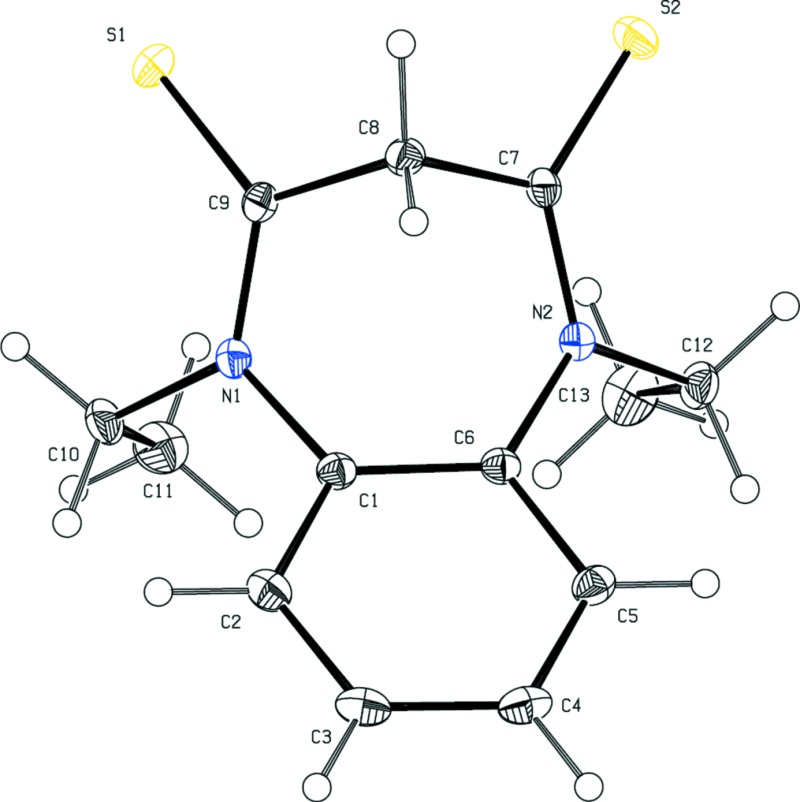
The structure of the title compound, showing atom labelling and 30% probability displacement ellipsoids.

. DOI: 10.1107/S205698901402790X/tk5353fig1.tif
The structure of the title compound, showing atom labelling and 30% probability displacement ellipsoids.

CCDC reference: 1040593

Additional supporting information:  crystallographic information; 3D view; checkCIF report

## Figures and Tables

**Table 1:** Hydrogen-bond geometry (, )

*D*H*A*	*D*H	H*A*	*D* *A*	*D*H*A*
C3H3S1^i^	0.95	2.86	3.6474(13)	141
C12H12*A*S1^ii^	0.99	2.87	3.4887(13)	121

Symmetry codes: (i) 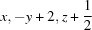; (ii) 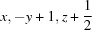.

## supplementary crystallographic information

### S1. Introduction

Benzodiazepines and their derivatives are an important class of bioactive compound. They have attracted attention of chemists in the field of pharmaceuticals (Kumar *et al.*, 2006). Some benzodiazepine derivatives have been widely used as anti-bacterial, anti-fungal, analgesic and anti-convulsant agents (Swamy *et al.* 2008).

### S2. Synthesis and crystallization

In a round flask, the 1,5 dietthyl benzodiazepine-2,4-diones (2,22 g, 10 ml) and P_2_S_5_ (4.44 g, 20 ml) were mixed in aceto­nitrile (50 ml). The mixture was refluxed for 4 h. After this time, the solvent was evaporated, and the residue formed was washed with HCl (2 N) solution and distilled water, dried and recrystallised from toluene-chloro­form (90/10). After some days, pale-yellow crystals were isolated (yield: 92.1 %, 2.34 g).

### S3. Refinement

Crystal data, data collection and structure refinement details are summarized in Table 1. H-atoms were placed in calculated positions (C—H 0.95–0.99 Å) and were included in the refinement in the riding model approximation, with *U*_iso_(H) = 1.5*U*_eq_(C) for methyl H atoms and 1.2*U*_eq_(C) for all other H atoms.

### S4. Results and discussion

The title compound crystallizes in the space group C2/c with one independent molecule in the asymmetric unit (Fig. 1). In the molecule, the diazepine ring system adopts a boat conformation with the two C1 and C6 atoms representing the stern and the C8 atom the prow with maximum deviation of 0.6626 (12)Å. The puckering parameters are: q2=0.9557 (11) Å, q3 = 0.2328 (11) Å, φ2 = 29.60 (7)° and φ3 = 128.4 (3)°. The mean plane of the diazepine ring is twisted with respect to that of the benzene ring by 32.27 (5)°. The geometric parameters of the title compound are comparable to those reported for similar structures (Ourahou *et al.*, 2010).

### Crystal data

**Table:**

C_13_H_16_N_2_S_2_	*F*(000) = 1120
*M**_r_* = 264.40	*D*_x_ = 1.321 Mg m^−^^3^
Monoclinic, *C*2/*c*	Mo *K*α radiation, λ = 0.71073 Å
Hall symbol: -C 2yc	Cell parameters from 7669 reflections
*a* = 19.8896 (2) Å	θ = 2.5–28.3°
*b* = 8.8743 (1) Å	µ = 0.38 mm^−^^1^
*c* = 15.5361 (2) Å	*T* = 150 K
β = 104.087 (1)°	Block, pale-yellow
*V* = 2659.75 (5) Å^3^	0.44 × 0.28 × 0.26 mm
*Z* = 8	

### Data collection

**Table:**

Bruker APEXII CCD area-detector diffractometer	3312 independent reflections
Radiation source: fine-focus sealed tube	2963 reflections with *I* > 2σ(*I*)
Graphite monochromator	*R*_int_ = 0.022
φ and ω scans	θ_max_ = 28.3°, θ_min_ = 2.1°
Absorption correction: multi-scan (*SADABS*; Bruker, 2009)	*h* = −26→26
*T*_min_ = 0.880, *T*_max_ = 0.906	*k* = −11→9
14634 measured reflections	*l* = −20→20

### Refinement

**Table:**

Refinement on *F*^2^	Primary atom site location: structure-invariant direct methods
Least-squares matrix: full	Secondary atom site location: difference Fourier map
*R*[*F*^2^ > 2σ(*F*^2^)] = 0.029	Hydrogen site location: inferred from neighbouring sites
*wR*(*F*^2^) = 0.083	H-atom parameters constrained
*S* = 1.05	*w* = 1/[σ^2^(*F*_o_^2^) + (0.0451*P*)^2^ + 1.6941*P*] where *P* = (*F*_o_^2^ + 2*F*_c_^2^)/3
3312 reflections	(Δ/σ)_max_ = 0.001
154 parameters	Δρ_max_ = 0.39 e Å^−^^3^
0 restraints	Δρ_min_ = −0.21 e Å^−^^3^

### Special details

**Table:**

Geometry. All e.s.d.'s (except the e.s.d. in the dihedral angle between two l.s. planes) are estimated using the full covariance matrix. The cell e.s.d.'s are taken into account individually in the estimation of e.s.d.'s in distances, angles and torsion angles; correlations between e.s.d.'s in cell parameters are only used when they are defined by crystal symmetry. An approximate (isotropic) treatment of cell e.s.d.'s is used for estimating e.s.d.'s involving l.s. planes.
Refinement. Refinement of *F*^2^ against ALL reflections. The weighted *R*-factor *wR* and goodness of fit *S* are based on *F*^2^, conventional *R*-factors *R* are based on *F*, with *F* set to zero for negative *F*^2^. The threshold expression of *F*^2^ > σ(*F*^2^) is used only for calculating *R*-factors(gt) *etc*. and is not relevant to the choice of reflections for refinement. *R*-factors based on *F*^2^ are statistically about twice as large as those based on *F*, and *R*- factors based on ALL data will be even larger.

### Fractional atomic coordinates and isotropic or equivalent isotropic displacement parameters (Å^2^)

**Table:**

	*x*	*y*	*z*	*U*_iso_*/*U*_eq_	
S1	0.389586 (17)	0.69948 (4)	0.221477 (18)	0.02667 (9)	
S2	0.401294 (17)	0.32244 (3)	0.37615 (2)	0.02698 (9)	
N1	0.38266 (5)	0.81930 (10)	0.37619 (6)	0.01868 (19)	
N2	0.38030 (5)	0.54640 (10)	0.48109 (6)	0.01790 (19)	
C1	0.39904 (5)	0.82159 (12)	0.47124 (7)	0.0180 (2)	
C2	0.41810 (6)	0.95882 (13)	0.51444 (8)	0.0236 (2)	
H2	0.4222	1.0462	0.4808	0.028*	
C3	0.43116 (6)	0.96826 (15)	0.60624 (8)	0.0270 (3)	
H3	0.4443	1.0618	0.6353	0.032*	
C4	0.42499 (6)	0.84098 (15)	0.65542 (8)	0.0258 (3)	
H4	0.4323	0.8482	0.7180	0.031*	
C5	0.40829 (6)	0.70361 (14)	0.61388 (7)	0.0217 (2)	
H5	0.4055	0.6164	0.6483	0.026*	
C6	0.39547 (5)	0.69179 (12)	0.52125 (7)	0.0176 (2)	
C7	0.41267 (5)	0.49358 (12)	0.42024 (7)	0.0183 (2)	
C8	0.45893 (6)	0.60552 (13)	0.38859 (7)	0.0196 (2)	
H8A	0.4898	0.6575	0.4396	0.024*	
H8B	0.4878	0.5541	0.3539	0.024*	
C9	0.41007 (5)	0.71650 (12)	0.33112 (7)	0.0185 (2)	
C10	0.32917 (6)	0.92648 (13)	0.32921 (8)	0.0255 (2)	
H10A	0.3380	0.9521	0.2709	0.031*	
H10B	0.3320	1.0205	0.3642	0.031*	
C11	0.25747 (7)	0.85946 (17)	0.31559 (9)	0.0341 (3)	
H11A	0.2230	0.9323	0.2846	0.051*	
H11B	0.2485	0.8353	0.3733	0.051*	
H11C	0.2544	0.7673	0.2801	0.051*	
C12	0.32830 (6)	0.45201 (14)	0.50965 (8)	0.0245 (2)	
H12A	0.3262	0.4814	0.5704	0.029*	
H12B	0.3427	0.3450	0.5112	0.029*	
C13	0.25706 (7)	0.46914 (18)	0.44750 (10)	0.0374 (3)	
H13A	0.2239	0.4054	0.4681	0.056*	
H13B	0.2588	0.4385	0.3875	0.056*	
H13C	0.2424	0.5746	0.4467	0.056*	

### Atomic displacement parameters (Å^2^)

**Table:**

	*U*^11^	*U*^22^	*U*^33^	*U*^12^	*U*^13^	*U*^23^
S1	0.03626 (18)	0.02858 (17)	0.01620 (14)	0.00172 (12)	0.00835 (11)	0.00226 (10)
S2	0.03522 (18)	0.01756 (15)	0.02775 (16)	0.00255 (11)	0.00685 (12)	−0.00303 (10)
N1	0.0206 (4)	0.0171 (5)	0.0179 (4)	0.0015 (3)	0.0039 (3)	0.0023 (3)
N2	0.0195 (4)	0.0165 (4)	0.0182 (4)	0.0003 (3)	0.0055 (3)	0.0014 (3)
C1	0.0159 (5)	0.0196 (5)	0.0186 (5)	0.0016 (4)	0.0044 (4)	−0.0005 (4)
C2	0.0233 (5)	0.0197 (5)	0.0279 (6)	−0.0003 (4)	0.0064 (4)	−0.0024 (4)
C3	0.0249 (6)	0.0270 (6)	0.0289 (6)	−0.0001 (5)	0.0062 (4)	−0.0108 (5)
C4	0.0221 (5)	0.0356 (7)	0.0196 (5)	0.0033 (5)	0.0047 (4)	−0.0066 (5)
C5	0.0193 (5)	0.0274 (6)	0.0191 (5)	0.0030 (4)	0.0059 (4)	0.0009 (4)
C6	0.0145 (5)	0.0192 (5)	0.0191 (5)	0.0019 (4)	0.0044 (4)	−0.0006 (4)
C7	0.0188 (5)	0.0180 (5)	0.0168 (4)	0.0043 (4)	0.0017 (4)	0.0031 (4)
C8	0.0181 (5)	0.0218 (5)	0.0198 (5)	0.0028 (4)	0.0063 (4)	0.0013 (4)
C9	0.0190 (5)	0.0185 (5)	0.0188 (5)	−0.0020 (4)	0.0065 (4)	0.0020 (4)
C10	0.0309 (6)	0.0198 (5)	0.0237 (5)	0.0078 (5)	0.0027 (4)	0.0046 (4)
C11	0.0255 (6)	0.0398 (8)	0.0349 (7)	0.0099 (6)	0.0035 (5)	0.0022 (6)
C12	0.0267 (6)	0.0230 (6)	0.0254 (5)	−0.0043 (5)	0.0097 (4)	0.0039 (4)
C13	0.0243 (6)	0.0473 (8)	0.0403 (7)	−0.0084 (6)	0.0071 (5)	0.0031 (6)

### Geometric parameters (Å, º)

**Table:**

S1—C9	1.6591 (11)	C5—H5	0.9500
S2—C7	1.6587 (11)	C7—C8	1.5150 (15)
N1—C9	1.3437 (15)	C8—C9	1.5131 (15)
N1—C1	1.4329 (13)	C8—H8A	0.9900
N1—C10	1.4803 (14)	C8—H8B	0.9900
N2—C7	1.3506 (14)	C10—C11	1.5118 (18)
N2—C6	1.4329 (14)	C10—H10A	0.9900
N2—C12	1.4805 (14)	C10—H10B	0.9900
C1—C2	1.3976 (15)	C11—H11A	0.9800
C1—C6	1.4008 (15)	C11—H11B	0.9800
C2—C3	1.3886 (17)	C11—H11C	0.9800
C2—H2	0.9500	C12—C13	1.5157 (17)
C3—C4	1.3858 (19)	C12—H12A	0.9900
C3—H3	0.9500	C12—H12B	0.9900
C4—C5	1.3815 (17)	C13—H13A	0.9800
C4—H4	0.9500	C13—H13B	0.9800
C5—C6	1.4030 (15)	C13—H13C	0.9800
			
C9—N1—C1	121.84 (9)	C9—C8—H8B	110.7
C9—N1—C10	120.88 (9)	C7—C8—H8B	110.7
C1—N1—C10	117.06 (9)	H8A—C8—H8B	108.8
C7—N2—C6	122.11 (9)	N1—C9—C8	114.72 (9)
C7—N2—C12	120.02 (10)	N1—C9—S1	124.58 (8)
C6—N2—C12	117.86 (9)	C8—C9—S1	120.56 (8)
C2—C1—C6	119.65 (10)	N1—C10—C11	110.88 (10)
C2—C1—N1	118.31 (10)	N1—C10—H10A	109.5
C6—C1—N1	122.03 (9)	C11—C10—H10A	109.5
C3—C2—C1	120.45 (11)	N1—C10—H10B	109.5
C3—C2—H2	119.8	C11—C10—H10B	109.5
C1—C2—H2	119.8	H10A—C10—H10B	108.1
C4—C3—C2	119.84 (11)	C10—C11—H11A	109.5
C4—C3—H3	120.1	C10—C11—H11B	109.5
C2—C3—H3	120.1	H11A—C11—H11B	109.5
C5—C4—C3	120.34 (11)	C10—C11—H11C	109.5
C5—C4—H4	119.8	H11A—C11—H11C	109.5
C3—C4—H4	119.8	H11B—C11—H11C	109.5
C4—C5—C6	120.55 (11)	N2—C12—C13	111.43 (10)
C4—C5—H5	119.7	N2—C12—H12A	109.3
C6—C5—H5	119.7	C13—C12—H12A	109.3
C1—C6—C5	119.09 (10)	N2—C12—H12B	109.3
C1—C6—N2	122.18 (9)	C13—C12—H12B	109.3
C5—C6—N2	118.72 (10)	H12A—C12—H12B	108.0
N2—C7—C8	115.48 (9)	C12—C13—H13A	109.5
N2—C7—S2	124.50 (9)	C12—C13—H13B	109.5
C8—C7—S2	119.93 (8)	H13A—C13—H13B	109.5
C9—C8—C7	105.35 (9)	C12—C13—H13C	109.5
C9—C8—H8A	110.7	H13A—C13—H13C	109.5
C7—C8—H8A	110.7	H13B—C13—H13C	109.5
			
C9—N1—C1—C2	131.40 (11)	C12—N2—C6—C5	47.51 (13)
C10—N1—C1—C2	−54.03 (14)	C6—N2—C7—C8	−7.63 (14)
C9—N1—C1—C6	−49.55 (15)	C12—N2—C7—C8	173.63 (9)
C10—N1—C1—C6	125.02 (11)	C6—N2—C7—S2	175.84 (8)
C6—C1—C2—C3	−2.23 (17)	C12—N2—C7—S2	−2.90 (14)
N1—C1—C2—C3	176.84 (10)	N2—C7—C8—C9	−71.66 (11)
C1—C2—C3—C4	−0.26 (18)	S2—C7—C8—C9	105.04 (9)
C2—C3—C4—C5	2.27 (18)	C1—N1—C9—C8	−0.07 (15)
C3—C4—C5—C6	−1.77 (17)	C10—N1—C9—C8	−174.43 (10)
C2—C1—C6—C5	2.70 (16)	C1—N1—C9—S1	175.67 (8)
N1—C1—C6—C5	−176.34 (10)	C10—N1—C9—S1	1.30 (15)
C2—C1—C6—N2	−176.18 (10)	C7—C8—C9—N1	77.46 (11)
N1—C1—C6—N2	4.78 (16)	C7—C8—C9—S1	−98.46 (10)
C4—C5—C6—C1	−0.73 (16)	C9—N1—C10—C11	87.54 (13)
C4—C5—C6—N2	178.20 (10)	C1—N1—C10—C11	−87.08 (12)
C7—N2—C6—C1	47.63 (15)	C7—N2—C12—C13	−85.53 (13)
C12—N2—C6—C1	−133.60 (11)	C6—N2—C12—C13	95.68 (12)
C7—N2—C6—C5	−131.26 (11)		

### Hydrogen-bond geometry (Å, º)

**Table:**

*D*—H···*A*	*D*—H	H···*A*	*D*···*A*	*D*—H···*A*
C3—H3···S1^i^	0.95	2.86	3.6474 (13)	141
C12—H12*A*···S1^ii^	0.99	2.87	3.4887 (13)	121

Symmetry codes: (i) *x*, −*y*+2, *z*+1/2; (ii) *x*, −*y*+1, *z*+1/2.
