# Supplementary figures and images for: Crystal structure of 1,5-diethyl-1H-1,5-benzodiazepine-2,4(3H,5H)-di­thione
Source: Acta Crystallogr E Crystallogr Commun. 2015 Jan 3;71(Pt 2):o83. doi: 10.1107/S205698901402790X (PMC4384617; doi:10.1107/S205698901402790X)

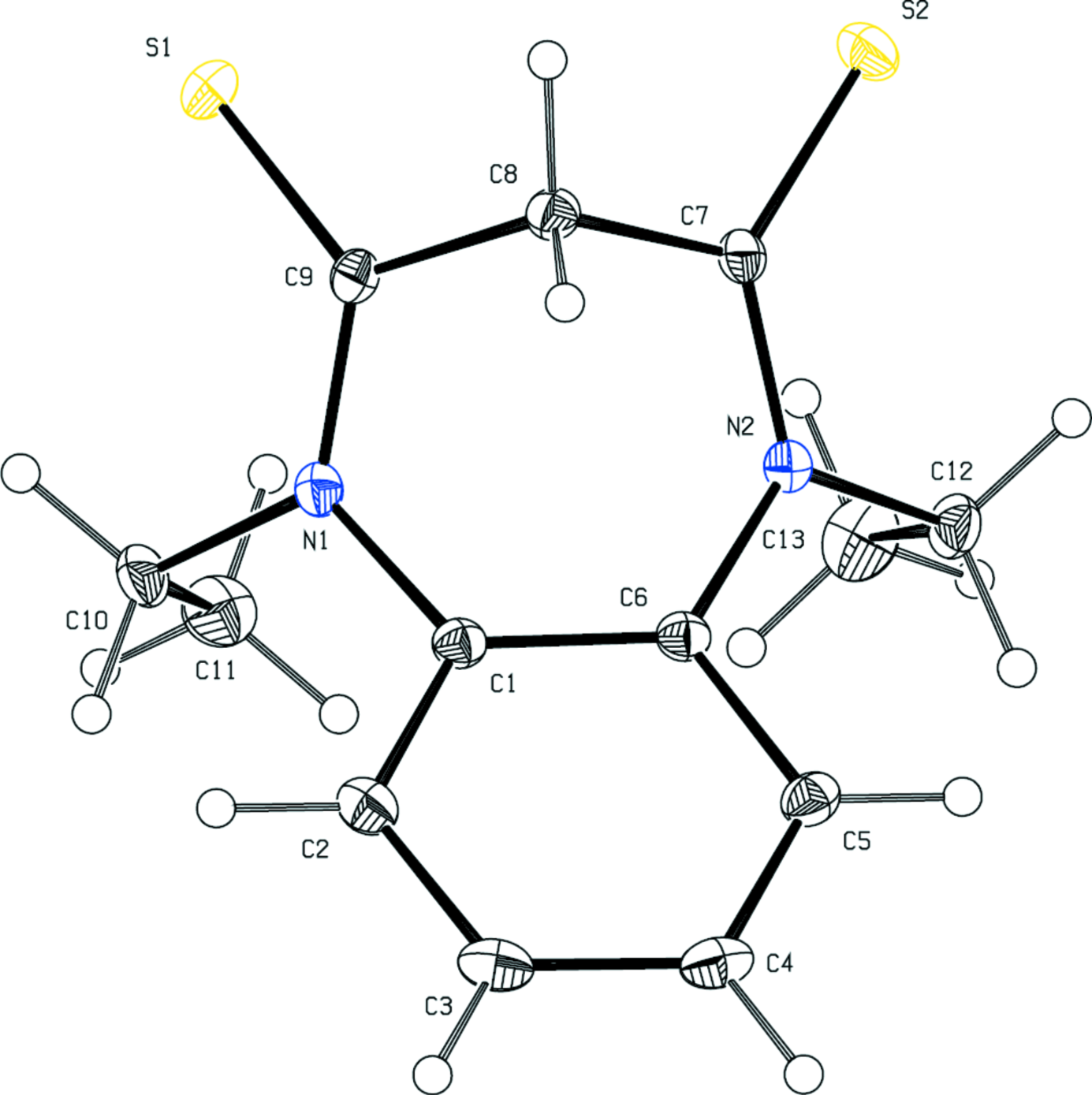

Supplement: Supplementary file 4 [file e-71-00o83-fig1.tif]
